# An Adjunct Indicator for the Diagnosis of Fracture-Related Infections: Platelet Count to Mean Platelet Volume Ratio

**DOI:** 10.7150/jbji.44116

**Published:** 2020-02-21

**Authors:** John Strony, Taylor Paziuk, Brianna Fram, Kyle Plusch, Gerard Chang, James Krieg

**Affiliations:** Rothman Orthopaedic Institute at Thomas Jefferson University, Philadelphia, PA. 19107, USA.

**Keywords:** Infection, Fracture, Platelet, Trauma, Nonunion

## Abstract

**Introduction:** Fracture-related infection (FRI) is a common complication associated with orthopaedic fracture care. Diagnosing these complications in the preoperative setting is difficult. Platelets are a known acute phase reactant with indices that change in accordance with infection and inflammation. The purpose of our study was to assess the diagnostic utility of platelet indices at assessing FRI.

**Methods:** A retrospective review performed for all patients who underwent revision surgery for fracture nonunion between 2013 and 2018. Radiographs were employed to define nonunion. Intraoperative cultures were used to define FRI. Receiver operator characteristic (ROC) curve analysis was used to assess the diagnostic ability of preoperative erythrocyte sedimentation rate (ESR), C-reactive protein (CRP), and the platelet count/mean platelet volume ratio (P/V) at recognizing FRI.

**Results:** Of the 53 revision surgeries that were performed for fracture nonunion, 17 (32.1%) were identified as FRI. There were no significant demographic differences between the two cohorts. Patients with FRIs exhibited higher values for ESR (54.82 vs. 19.16, p<0.001), CRP (0.90 vs. 0.35, p=0.003), and P/V (37.4 vs. 22.8, p<0.001) as compared to those within the aseptic nonunion cohort. ROC curve analysis for P/V demonstrated that at an optimal ratio of 23, area under the curve (AUC) is 0.814, specificity is 55.6%, and sensitivity is 100.0%. There was no significant difference in the diagnostic performance of the serum biomarkers but only ESR and P/V had an AUC greater than 0.80. The negative predictive value (NPV) for P/V, ESR, and CRP was 100.0%, 84.6%, and 78.6%, respectively.

**Conclusion:** The P/V ratio may serve as a reliable screening test for FRI.

## Introduction

Fracture nonunion remains a commonly encountered problem within the United States despite advances in fracture management 1,2. The complications can be a serious source of morbidity for patients due to the possibility of multiple revision surgeries, extensive healing time, and functional impairment. These complications can arise via several different mechanisms, including mechanical issues such as inadequate reduction or excessive motion at the fracture site secondary to insufficient fixation, or biological issues such as endocrinopathies, inadequate blood supply, nutritional deficiency, or infection 16. The latter, termed septic nonunion or fracture-related infection (FRI); provides an additional layer of complexity in the assessment and management of patients. While overt clinical exam findings like purulent drainage is a widely accepted way of diagnosing FRI, patients rarely present with these definitive preoperative findings and are therefore left with intraoperative cultures as their definitive diagnostic strategy 11,15,17. Preoperative serum markers like white blood cell count (WBC), erythrocyte sedimentation rate (ESR), and C-reactive protein (CRP) also play an important role in the diagnostic evaluation of these patients 2,11,17,18. Nonetheless, several studies have demonstrated that these markers lack the diagnostic ability to reliably screen patients for potential FRI 2*.* Therefore, there exists a significant demand for an efficient preoperative screening test in this cohort.

There are numerous studies that have extensively described the role that platelets play in our bodies inflammatory response to bacterial invasion 6,12,20. In addition, there have been a handful of studies that have investigated the potential role that the indices we use to measure platelets may play in the diagnostic evaluation of a variety of inflammatory and infectious states 1,4,7,21. However, there is a paucity of literature investigating these indices, specifically when measured as a ratio (P/V), in the realm of orthopaedic surgery and more importantly FRI. Therefore, the purpose of this study was to determine whether or not two commonly ordered lab values that have well documented changes in accordance with acute states of inflammation and infection, specifically platelet count (PC) and mean platelet volume (MPV), could further aid in the diagnostic evaluation of patients with suspected FRI 6, 12, 20. We hypothesize that platelet indices will change in accordance with FRI and will therefore help guide orthopedic surgeons in the assessment of patients with fracture non unions.

## Methods

After institutional review board (IRB) approval, we conducted a retrospective review of all fracture nonunion revision surgeries performed by a single fellowship trained orthopaedic trauma surgeon at a single institution between 2013 and 2018. Eligibility criteria included all patients undergoing revision surgery for a nonunion. We defined nonunion as an arrest in the biologic fracture repair process, as seen on imaging, for three consecutive months with a minimum of nine months between the index procedure and diagnosis 2. In addition, all included patients had cultures taken at the time of revision surgery. Such cultures involved obtaining multiple samples at the nonunion site and adjacent tissue. We defined a nonunion to be infection related if one or more intraoperative cultures were positive. If the only positive culture was isolated from broth, then the nonunion was deemed aseptic. Finally, patients needed documentation of preoperative ESR, CRP, and complete blood count (CBC). Any patient who did not have a recorded PC and/or MPV as part of their CBC was excluded. Based on the results of their intraoperative cultures, the patient population was divided into septic and aseptic nonunion cohorts.

All blood tests were performed in the laboratories of Thomas Jefferson University Hospital. The PC and MPV were calculated via a Sysmex XN analyzer. Impedance measurements that employ the Coulter principle were used to calculate both PC and MPV. Both impedance measurements are derived from the impedance platelet size distribution. The Sysmex analyzer is able to generate an accuracy flag for irregular samples. This accuracy flag causes the samples to be reflexed to a fluorescent channel for staining and flow cytometry analysis of forward and side scatter to derive PC and MPV. The P/V ratio was calculated by using the following formula:


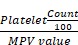


ESR was presented as a mean with standard deviations. Because of their non-normal distribution, the P/V ratio and CRP were presented as a median and interquartile range. The units used for ESR and CRP were mm/hr and mg/dL, respectively. Fisher exact test, Independent T-test or Mann-Whitney U test was used to define statistical significance with a cutoff of P<0.05. For each parameter, true positive, true negative, false positive and false negative results were calculated. Sensitivity and specificity as well as the positive predictive value (PPV) and negative predictive value (NPV) were calculated. The optimal diagnostic performance and diagnostic utility of the serum biomarkers was assessed using receiver operator characteristic (ROC) curve analysis. The diagnostic performance of the serum biomarkers was quantified as the corresponding area under the curve (AUC). Finally, through the use of ROC curves, the diagnostic performance of all three values was combined and subsequently compared to the performance of the P/V ratio alone and the combination of ESR and CRP. All statistical analyses were performed with use of MedCalc Statistical Software.

## Results

Overall, 120 revision surgeries for fracture nonunion were performed between 2013 and 2018. Ninety-two (76.7%) of these surgeries included intraoperative cultures, but only 53 (57.6%) had documentation confirming at least 3 intraoperative cultures taken proximal, distal, and at the site of nonunion. Lower extremity fractures occurred in 48 (90.6%) of the remaining 53 patients. Of these fractures, 27 (56.3%) occurred below the knee and 21 (43.7%) occurred above the knee. Nineteen of the 53 (35.8%) patients had fractures that were deemed open prior to their index procedure, but only 7 (36.8%) of the 19 developed a FRI. Seventeen (32.1%) of the 53 surgeries had at least one positive intraoperative culture while 36 (67.9%) had negative cultures. The median number of positive intraoperative cultures was 2 (range, 1-7 positive cultures). There were seven patients with only one positive intraoperative culture, of which none of these patients had a positive culture that was only isolated from broth and all of the 7 were treated for a FRI based on intraoperative assessment and discussions with our infectious disease consultants. The two most commonly isolated organisms were Staphylococcus *aureus* (6/17; 35.3%) and Staphylococcus *epidermidis* (6/17; 35.3%). In the cohort of patients that only had 1 positive culture, the most commonly isolated organism(s) was Staphylococcus *epidermidis* or Coagulase negative Staphylococcus (3/7; 42.9%), of which, 1 also isolated Enterobacter *cloacae* from the same specimen. The remaining 4 single positive cultures patients isolated Staphylococcus *aureus* (2/7; 28.6%) or Enterobacter cloacae (2/7; 28.6%). None of the 53 patients included in this study met the definition of systemic inflammatory response syndrome (SIRS) preoperatively, defined as having two or more of the following clinical criteria: temperature greater than 38 or less than 36 degrees Celsius, WBC count greater than 12 or less than 4, heart rate above 90 beats per minute, or a respiratory rate greater than 20 breaths per minute 3,5. Therefore, no patient included in our study received preoperative antibiotics for a presumed diagnosis of FRI. The study population was predominantly female (n=31) with a mean age of 51.5 years (range, 17-78 years). Fractures of the tibia were the most prevalent injury (n=26), followed by the femur (n=21). Sixteen of the original injuries were open fractures. There was no significant difference between the two cohorts with regards to age, Charlson Comorbidity Index (CCI), Body Mass Index (BMI), smoking status, alcohol use, and pre-admission medications associated with changes in platelet indices (Table 1). In addition, no patients in either cohort had a history of human immunodeficiency virus (HIV), Hepatitis C virus, or cirrhosis.

Serum marker details are provided in Tables 2 and 3.

Patients with fracture related infections exhibited higher values for ESR, CRP and P/V ratios as compared to those with aseptic nonunion. The mean ESR was 54.8 (standard deviation (SD) = 34.4) in the FRI group vs. 19.2 (SD = 14) in the aseptic group (p = 0.001). The median CRP was 0.9 vs. 0.35 (p=0.003) and the median P/V ratio was 37.4 vs. 22.8 (p<0.001) for patient with septic and aseptic nonunions, respectively.

**Figure 1:**
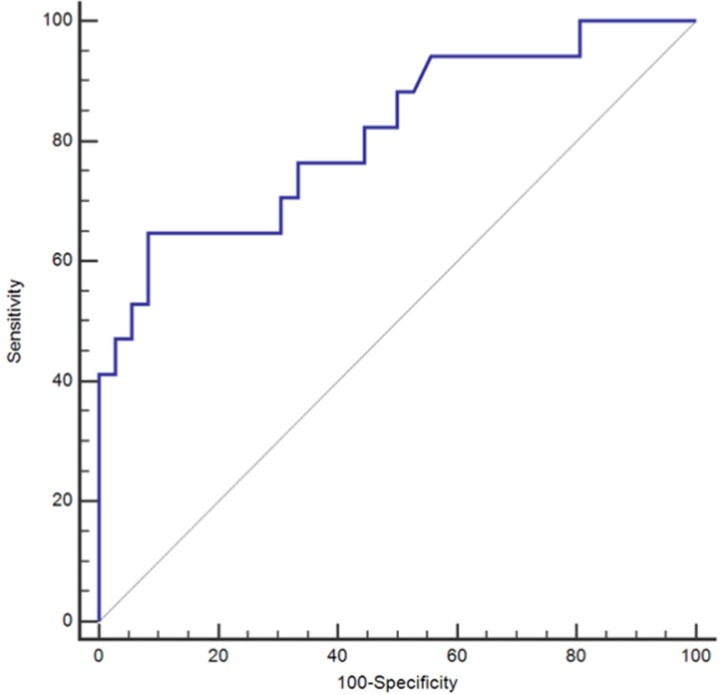
Receiving Operating Characteristic Curve (ROC) for Platelet Count to Mean Platelet Volume (P/V) Ratio alone; *ROC =Receiver Operating Characteristic.

For the P/V ratio, the area under the curve (AUC) of the ROC curve analysis was 0.814. At an optimal ratio of 23, the sensitivity of this test would be 100.0% (95% CI 80.5-100.0) with a corresponding specificity of 55.6% (95% CI, 38.1-72.1%). At this ratio, the negative predictive value (NPV) was 100.0% and positive predictive value (PPV) 51.5% (95%CI, 42.4-60.5%) (Table 2) (Figure 1). Compared to the P/V ratio, both ESR and CRP yielded numerically greater levels of specificity at the cost of sensitivity with the utilization of the optimal values defined by the ROC curve analysis (Table 2). The ROC curve analysis for ESR yielded an AUC of 0.813 (Table 2). At an optimal threshold of 35, ESR demonstrated a sensitivity of 64.7%, specificity 91.7%, NPV 84.6%, and PPV 78.6% (Table 2). For CRP, ROC curve analysis provided an AUC of 0.752, while yielding a sensitivity of 47.1%, specificity 91.7%, NPV 78.6%, and PPV 72.7% at an optimal value of 1.2 (Table 2). Overall, the diagnostic accuracies of the three individual markers were similar and not significantly different from each other despite the fact that only the P/V ratio and ESR reached a diagnostic AUC of greater than 0.8 (Table 2).

**Figure 2:**
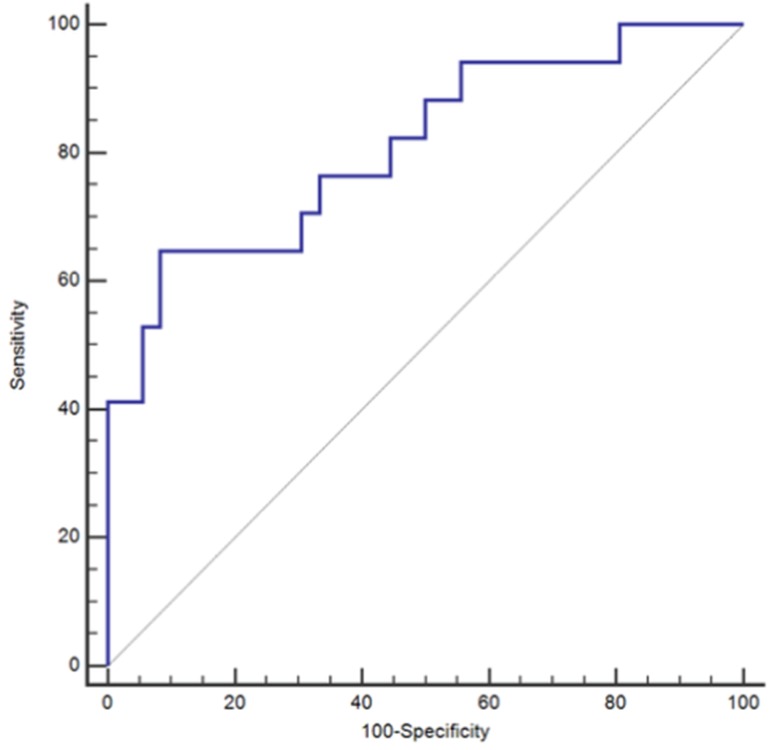
Receiving Operating Characteristic Curve (ROC) for Erythrocyte Sedimentation Rate (ESR) and C-Reactive Protein (CRP) when used in combination; *ROC = Receiver Operating Characteristic.

**Figure 3:**
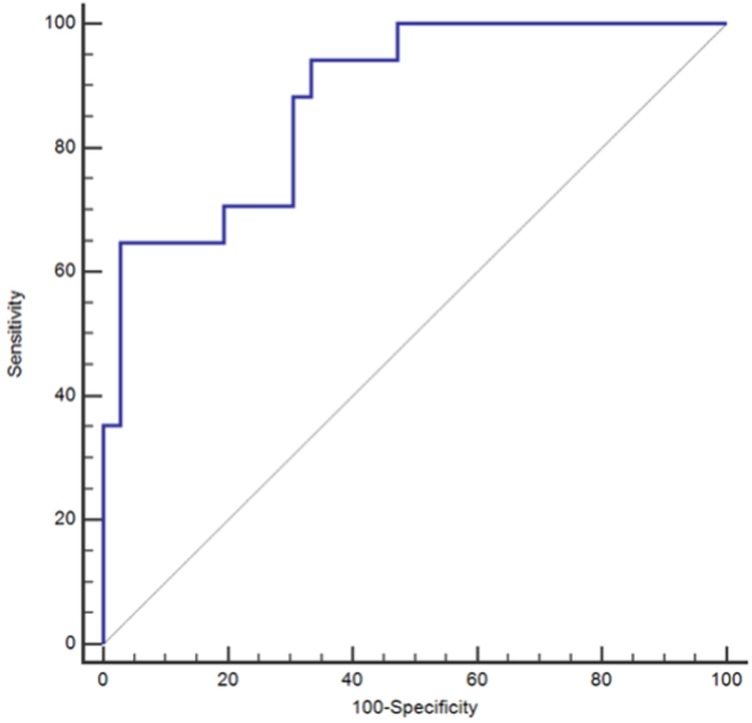
Receiving Operating Characteristic Curve (ROC) for Erythrocyte Sedimentation Rate (ESR), C-Reactive Protein (CRP), and the Platelet Count to Mean Platelet Volume (P/V) Ratio when used in combination; *ROC = Receiver Operating Characteristic.

Additional ROC curve analyses were conducted to examine the performance of ESR and CRP in conjunction with each other as well in conjunction with the P/V ratio. The merging of ESR with CRP resulted in no increased performance of the paired biomarker as compared to that of ESR alone. An ROC curve analysis yielded an AUC of 0.81 with corresponding sensitivity of 64.7% and specificity of 91.7% (Table 3) (Figure 2). When all three markers were combined there was a non-significant (p=0.0814) numeric increase of the diagnostic performance of the analysis with a resulting AUC of 0.879 and a corresponding sensitivity of 64.7%, specificity 97.2%, NPV 85.4%, and PPV 91.7% (Table 3)(Figure 3).

## Discussion

FRI remains a devastating complication associated with fracture care in the United States. ESR and CRP have traditionally been used preoperatively to assess patients for potential FRI. However, due to the lack of diagnostic reliability associated with these serum biomarkers, the gold standard for diagnosis is still intraoperative cultures. In this retrospective review we found that platelet indices, when measured as the P/V ratio, may be a more reliable screening test relative to the more traditional serum biomarkers.

The ideal serum biomarker identifies all patients with or without a given disease such that an accurate diagnosis can be made without any further invasive testing. However, such a circumstance rarely exists, and therefore the primary function of most serum biomarkers is screening, such that they act as a type of sorting mechanism for identifying patients who require further diagnostic testing. To do so, serum biomarkers should correctly identify all individuals who have a given disease while allowing only a modest number of patients without the disease to be incorrectly labeled as having the disease. In this retrospective analysis, we studied the clinical utility of the P/V ratio as a potential diagnostic tool in the context of FRIs. We compared this ratio to the more tradetional inflammatory biomarkers ESR and CRP. Platelet indices were chosen for multiple reasons. First, they are widely accessible because most patients should receive a preoperative complete blood count (CBC). Second, platelets are an acute phase reactant indices change in accordance with infectious and inflammatory processes. We decided to employ the ratio of the PC and MPV in our analysis because several studies have demonstrated that PC increase while MPV decreases during reactive thrombocytosis, which creates a larger P/V ratio and potentially accounts for patients with baseline thrombocytosis 1,7,8,21.

Although platelets were traditionally thought of as only an ancillary player in our bodies response to infection, primarily via the synthesis and secretion of inflammatory cytokines like interleukin-1 (IL-1), interleukin-6 (IL-6), and tumor necrosis factor (TNF), recent literature demonstrate that they play a much more active role than previously thought 8,9,19,20. Platelets are not only actively recruited to sites of inflammation and infection, but they are capable of fighting pathogens directly through the use of antimicrobial compounds and/or physical trapping 19,20.

Our analysis reinforces the idea that ESR and CRP do not exhibit sufficient sensitivity or specificity for reliably predicting the presence or absence of FRIs. Even when the threshold for a positive test is set at its optimal value as defined by ROC curve analysis, ESR and CRP only demonstrated sensitivities of 64.7% and 47.1%, respectively. Based on these values, there is a significant risk for false negatives with these tests. In contrast, with a NPV of 100.0%, the P/V ratio may serve as a more useful clinical tool as it could reliably rule out an infectious etiology for a given patient's fracture nonunion. However, further prospective studies are needed to refine and characterize this biomarker.

There were several limitations associated with our retrospective study design. First, the way in which our blood samples were collected and stored was not standardized between patients and therefore subjects our platelet indices to measurement errors 13. Although there was no way to account for these pre-analytical variables in our retrospective study design, the fact that all of our samples were assessed using the same Sysmex analyzers minimized any further variability in how the platelet indices were recorded 10. The second limitation associated with this study design was that the analyzable population was from a single institution and therefore represents a limited geographic area. This limitation may impact the generalizability of our result to a larger and more geographically diverse population. In addition, because our population is small, our study was likely underpowered and therefore may subject our results to type-2 errors. The third limitation associated with this retrospective study design is that our results are subject to selection bias, as there could have reasonably been confounding variables that could not be quantified outside of the patient demographics listed in Table 1. The fourth limitation of this study pertains to the way we defined FRI, as 7 patients in the FRI cohort had only 1 positive culture. While this represents a deviation from the standard definition, only 2 of these cases grew possible contaminant organisms like S. epidermidis or Coagulase-Negative Staph in isolation, and all patients within this cohort were treated for FRI based on clinical assessment. Therefore, because we clinically deemed them infected and the purpose of our assessment was to provide a more sensitive biomarker, the decision was made to include these patients in the FRI cohort. Lastly, although intraoperative cultures are considered the gold standard for diagnosing FRI, Palmer et al. demonstrated that the accuracy of cultures is limited secondary to their low sensitivity 14. While this is undoubtedly a difficult issue, as it further predisposes our results to type-2 errors, it only functions to highlight the differences we did see between the two cohorts, as there could have reasonably been patients in the aseptic cohort who really did have an FRI despite negative cultures.

Our results show that the P/V ratio may be a reliable screening test for FRI. Therefore, we recommend that surgeons begin to evaluate platelet indices, and more specifically the P/V ratio, as a potential screening tool for their patients with potential FRIs. Larger prospective trials are needed in the future to further elucidate the utility of the P/V ratio in the perioperative management of patients with FRI.

## Figures and Tables

**Table 1:** Patient demographics

	FRI (N=17)	Aseptic (N=34)	P Value
CCI (median)	1.0	1.0	0.979
BMI (median)	25.8	31.0	0.137
Age* (years)	51.1	51.7	0.879
Gender (Female)	64.7%	55.6%	
Smoking Status (Positive History)	35.3%	25.0%	0.520
Alcohol Status (Positive History)	41.2%	52.8%	0.559
Medications**	76.5%	66.7%	0.538

* Reported as Mean. **Patients on Medications that list thrombocytosis or thrombocytopenia as possible “Rare” adverse effects. *** FRI = Fracture Related Infection; CCI = Charlson Comorbidity Index; BMI = Body Mass Index.

**Table 2:** Receiving Operating Characteristic Curve (ROC) Analysis of Serum Biomarkers in the Diagnosis of Fracture-Related Infection

	P/V Ratio	ESR (mm/hr)	CRP (mg/dL)
Optimal Threshold	23.0	35.0	1.2
Area Under the Curve (AUC)	0.814	0.813	0.752
Specificity (95% CI)	55.6% (38.1-72.1)	91.7% (77.53-98.25)	91.7% (77.53-98.25)
Sensitivity (95% CI)	100.0% (80.5-100)	64.7% (38.3-85.8)	47.1% (22.98-72.19)
Accuracy	69.8%	83.0%	77.4%
Positive Likelihood Ratio	2.3	7.8	5.7
Negative Likelihood Ratio	0.0	0.4	0.6
Positive Predictive Value	51.5%	78.6%	72.7%
Negative Predictive Value	100.0%	84.6%	78.6%
Standard Error	0.0111	0.0674	0.0735
95% Confidence Interval	0.683-0.907	0.682-0.907	0.615-0.861
Z-Statistic	5.420	4.645	3.433
Significance	<0.001	<0.001	0.006

*P/V = Platelet Count to Mean Platelet Volume Ratio; ESR = Erythrocyte Sedimentation Rate; CRP = C-Reactive Protein; CI = Confidence Interval.

**Table 3:** A Comparison of the Diagnostic Performance of Two Models for Diagnosing Fracture Related Infection

Test	ESR + CRP	ESR + CRP + P/V Ratio	P-Value
Area Under the Curve (AUC)	0.810	0.879	0.0814
Specificity	91.7% (77.5-98.3)	97.2% (85.5-99.9)	
Sensitivity	64.7% (38.3-85.8)	64.7% (38.3-85.8)	
Accuracy	83.0%	86.8%	
Positive Likelihood Ratio	7.8	23.3	
Negative Likelihood Ratio	0.4	0.4	
Positive Predictive Value	78.6%	91.7%	
Negative Predictive Value	84.6%	85.4%	

*P/V = Platelet Count to Mean Platelet Volume Ratio; ESR = Erythrocyte Sedimentation Rate; CRP = C-Reactive Protein.
